# Apoptosis and turnover disruption of olfactory sensory neurons in eosinophilic chronic rhinosinusitis

**DOI:** 10.3389/fncel.2024.1371587

**Published:** 2024-02-28

**Authors:** Yuetong Chen, Minghan Li, Juan Lu

**Affiliations:** ^1^The First School of Clinical Medicine, Southern Medical University, Guangzhou, China; ^2^Department of Otorhinolaryngology Head and Neck Surgery, Nanfang Hospital, Southern Medical University, Guangzhou, China

**Keywords:** olfactory sensory neurons, apoptosis, turnover, eosinophils, chronic rhinosinusitis, olfactory dysfunction

## Abstract

Olfactory dysfunction (OD) is one of the important and difficult-to-treat symptoms of eosinophilic chronic rhinosinusitis (CRS), which is typically associated with type 2 inflammation where eosinophils (EOSs) function as both effectors and initiators. Eosinophilic infiltration in the olfactory mucosa (OM) is associated with severe OD, mucosal erosion, and more loss of olfactory sensory neurons (OSNs). Active EOS-derived cytokines, chemokines, and eosinophil granule proteins may lead to aggravation of inflammation, tissue damage, and impairment of the survival and regeneration of OSNs. Recent studies show that EOSs can lead to apoptosis of OSNs through axonal and neural body damage, turnover disorder of OSNs through the loss of immature OSNs and globose basal cells (GBCs), changed proliferative activity of horizontal basal cells (HBCs), and dysfunction of OSNs through the breakdown of neuroepithelial integrity and alteration of ion concentration in OSNs and mucin. In this review, we outline the current progress on the role of EOSs on OD in patients with eosinophilic CRS and the mechanism of EOS-associated injury of the OM and OSNs in experimental animal models with sinonasal inflammation. Further investigations on the molecular mechanisms of tissue eosinophilia-induced injury of OSNs are warranted to obtain new therapeutic targets and achieve better restoration of olfactory function.

## Introduction

Eosinophils (EOSs) are known to play multiple roles in immune maintenance, host defense against bacterial and viral infections, immune regulation through T-helper 1 (Th1)/T-helper 2 (Th2) balance modulation, and the aggravation of inflammation and tissue damage. In response to various stimuli, EOSs release a variety of mediators, including cytokines, chemokines, lipid mediators, enzymes, and eosinophil granule proteins. In the sinonasal mucosa of patients with chronic rhinosinusitis (CRS), eosinophilic infiltration induces epithelial cell disruption, basal cell and goblet cell hyperplasia, mucin hypersecretion, collagen deposition in the basement membrane, and epithelial–mesenchymal transition, which may promote nasal polypogenesis. EOSs are more connected to chronic rhinosinusitis with nasal polyps (CRSwNP) than chronic rhinosinusitis without nasal polyps (CRSsNP). Patients with eosinophilic chronic rhinosinusitis (ECRS) have more severe inflammation of the sinonasal mucosa than those with non-eosinophilic chronic rhinosinusitis (nECRS). ECRS is typically associated with type 2 inflammation, where EOSs function as both effectors and initiators (Bochner and Stevens, 2021; Kim et al., 2023).

Olfactory dysfunction (OD), including hyposmia and anosmia, is a common condition in the general population, whose reported prevalence ranges from 4 to 25% and has a significant impact on emotional and mental health and quality of life (Keller and Malaspina, 2013). Among various causes of OD, sinonasal disease was the most common cause before the coronavirus disease 2019 (COVID-19) pandemic and is now the second most common cause, next only to upper respiratory infection (Whitcroft et al., 2023). Rhinosinusitis is the main cause of OD due to sinonasal disease. Although OD is one of the important and difficult-to-treat symptoms of CRS and presents in 60% to 84% of patients with CRS, OD secondary to CRS is more likely to recover compared to other causes (Mattos et al., 2019; Fokkens et al., 2020; Smith et al., 2021; Lin and Yeh, 2022). Eosinophilic infiltration in the sinonasal mucosa is associated with not only refractory CRS but also severe OD and is a negative predictor of olfactory outcome after endoscopic sinus surgery (ESS). This narrative review aimed to outline current progress on the effects of EOSs on the injury of olfactory sensory neurons (OSNs) through an electronic search on PubMed and Medline.

## EOSs and OD secondary to CRS

OD in patients with CRS has overlapping etiologies, including a conductive mechanism due to nasal polyps, discharge, mucosal edema, and mucociliary clearance impairment in the nasal cavity and olfactory cleft (OC), blocking odorant transmission to the olfactory mucosa (OM), a sensorineural mechanism due to inflammation of the OM and consequent morphological and functional damage of OSNs, and a central mechanism due to resultant apoptosis of relay neurons and volume reduction of the olfactory bulb (OB) and olfactory brain gray matter (Rombaux et al., 2008; Yee et al., 2010; Han et al., 2017). A meta-analysis of clinical studies of olfactory function in CRS populations shows that OD is more prevalent in patients with CRSwNP than in those with CRSsNP (Kohli et al., 2017).

Among various infiltrating inflammatory cells in sputum, nasal polyps, and sinonasal mucosa, EOSs are most significantly correlated with the severity of CRS and nasal polyps (Thompson et al., 2016; Kanemitsu et al., 2020). Patients with highly activated EOSs in the ethmoid sinus are more likely to report olfactory loss in addition to nasal obstruction, nasal discharge, and headache, which are the other three main symptoms of CRS (Haruna et al., 2006). However, recent studies show that EOSs in OM correlate better with OD than other sinonasal tissues (Wu et al., 2020; Farrell et al., 2021). In the superior turbinate tissue of patients with CRSwNP, elevated gene expression of the EOS marker Charcot-Leyden crystal protein is positively correlated with interleukin-5 (IL-5) but negatively correlated with olfactory function (Lavin et al., 2017). OD in patients with CRSwNP is particularly associated with type 2 cytokine levels in OC mucus and OM (Soler et al., 2020; Gomes et al., 2023). Levels of Galectin-10, a major constituent of human EOSs, in both OC mucus and OM, are positively correlated with tissue eosinophilic counts but negatively correlated with olfactory function (Liu et al., 2023).

Tissue eosinophilia and EOS activation are associated with OD in ECRS, independent of polyps and disease severity (Haruna et al., 2006; Hauser et al., 2017; Suzuki et al., 2022). Patients with ECRS, regardless of nasal polyps, aspirin-exacerbated respiratory disease, or central compartment atopic disease, have a more severe OD than those with nECRS (Laidlaw et al., 2021; Lin and Yeh, 2022). Endotypes defined with eosinophilic infiltration and type 2 cytokines in OC are more effective at predicting OD than phenotypes defined with visible polyps on endoscopy. Eosinophilic infiltration rather than inflammation in the postoperative ethmoid sinus mucosa may predict negative olfactory improvement after ESS (Zhang et al., 2019). A recent study shows that reboot surgery can improve olfactory function for at least 6 months in patients with ECRSwNP by completing the removal of inflamed mucosa in the ethmoid sinus (Gomes et al., 2023).

OD is a hallmark symptom for patients infected with severe acute respiratory syndrome coronavirus 2 (SARS-CoV-2; Desai et al., 2022). In contrast to the role of EOSs in CRS, eosinopenia is not only an early biomarker of COVID-19 but also a risk factor for severe outcomes of COVID-19, including anosmia and ageusia (Sehanobish et al., 2021). Angiotensin-converting enzyme 2 (ACE2) and transmembrane serine protease 2 (TMPRSS2), receptors targeted by SARS-CoV-2 to bind and enter cells, are highly expressed in the sinonasal mucosa and OM (Ziegler et al., 2020). EOSs and Th2 cytokines can reduce ACE2 and TMPRSS2 mRNA expression and inhibit SARS-CoV-2 variants, which may decrease the risk of ECRS patients for COVID-19 and an uncontrolled inflammatory response. This means a decreased risk for ongoing OM injury, olfactory function damage, and virus entry into the OB and central nervous system (Marin et al., 2021; Macchia et al., 2023). On the contrary, ACE2 and TMPRSS2 gene expressions are upregulated by Th1 cytokines such as interferon-γ and tumor necrosis factor α (TNF-α), increasing the susceptibilities of nECRS patients to COVID-19 and further inflammation in OM and OD (Chen et al., 2020; Kawasumi et al., 2022).

## EOSs and pathological changes in OM

OM is composed of neuroepithelium, basement membrane, and lamina propria. Olfactory neuroepithelium (ONE) contains OSNs, sustentacular cells, microvillar cells, and basal cells that are located close to the basement membrane. Lamina propria in the subepithelial layer contains unmyelinated axons projecting from OSNs to the olfactory bulb (OB), olfactory ensheathing cells, Bowman’s glands with ducts that extend to the ONE, lymphocytes, and blood vessels.

In patients with CRS, there are four typical types of histopathological changes in ONE: normal pseudostratified, goblet cell hyperplasia, squamous metaplasia, and epithelial erosion. In lamina propria, there are morphological alterations, such as thickening of the basement membrane, edema, and increased presence of EOSs, neutrophils, lymphocytes, mast cells, and macrophages. Inflammation in the OM is particularly evident in the lamina propria in CRS patients with severe OD. OSNs have normal morphology in normal pseudostratified and goblet cell hyperplasia but have abnormal morphology in squamous metaplasia. In epithelial erosion, there are various degrees of mucosal damage, and the severity of morphological damage is positively correlated with the degree of immune cell infiltration and loss of OSNs (Kern, 2000). It is reported that in all CRS patients with OD, OM exhibits moderate to severe inflammatory changes and morphological damage, which are positively correlated with the degree of inflammatory cell infiltration and severity of OD (Van Zele et al., 2006; Yee et al., 2009).

Studies show that EOSs play a unique role in histopathological and functional changes in inflamed OM. Patients with ECRS have more EOSs, greater epithelial erosion, fewer OSNs in OM, and lower olfactory scores and worsening of OD symptoms after ESS than those with nECRS (Kashiwagi et al., 2019; Ahn et al., 2020). Studies on superior turbinate mucosa in patients with CRS with or without polyps show that only EOSs, rather than other inflammatory cells, are elevated in OM, have an adverse impact on the integrity of OM, and are correlated with squamous metaplasia, mucosal erosion, loss of OSNs, and severity of OD (Yee et al., 2010; Lavin et al., 2017).

## Mechanisms of EOS-induced OSN damage

EOSs are directly related to neuropathology. Major basic protein (MBP) and eosinophil peroxidase (EPO) released by active EOSs are selective allosteric antagonists of the M2 muscarinic receptors, which may enhance vagus-mediated bronchoconstriction in asthma (Jacoby et al., 1993). Eosinophilic infiltration is associated with focal axonal loss and capillary dilatation in peripheral nerves in Churg–Strauss syndrome and peripheral neuropathy in eosinophilic granulomatosis with polyangiitis (Oka et al., 2011; Chen et al., 2023). Inflammation of the OM can affect neurogenesis, differentiation, and maturation of OSNs (Lin and Yeh, 2022). OSNs and their progenitors are particularly susceptible to local immune mediators (Lane et al., 2010). EOS-released eosinophilic cationic protein (ECP), MBP, and β-glucuronidase induce apoptosis of OSNs, and their concentrations are quantitatively related to OD (Becker et al., 2012). Neurotoxic products released from active EOSs can damage OM and affect the survival and regeneration of OSNs (Acharya and Ackerman, 2014; Akasheh et al., 2014; Feng et al., 2019).

EOS-associated pathological changes in OM observed in patients with CRS have been validated with experimental murine models of allergic CRS (Rouyar et al., 2019), eosinophilic CRS (Kagoya et al., 2021), CRSwNP induced by ECP (Kikuta et al., 2021) and IL-4 nasal drops (Hara et al., 2023), conditional expression of IL-13 in OSNs (Saraswathula et al., 2023), and allergic rhinitis (Epstein et al., 2008; Ozaki et al., 2010; Carr et al., 2012; Selvaraj et al., 2012; Sousa Garcia et al., 2017). The results of these studies are summarized in Table 1.

**Table 1:** Murine models with eosinophilic infiltration in OM.

Study	Model	Inducer	ONE	Lamina propria	Olfactory cleft	Olfactory function
Rouyar et al. (2019)	Allergic CRS	House dust mite and *Staphylococcus aureus* enterotoxin B	Infiltration of mast cells. imOSN and GBC number loss. Slightly HBC number increase.	Infiltration of EOSs and mast cells. EOSs near the nerve bundle. Increased IL-13, IL-14, and IL-5 mRNA level. Increased IL-4, IL-5, IL-13, TSLP, IL-13 receptor subunits, and IL-10 Protein level.	-	Not changed.
Kagoya et al. (2021)	ECRS	MC903 and ovalbumin	Thickness reduction. mOSN number loss.	-	-	-
Kikuta et al. (2021)	CRSwNP	ECP	Thickness reduction. imOSNs and mOSNs number loss. OSNs detached from the basal lamina and apoptosis. Some basal cells apoptosis.	-	-	-
Hara et al. (2023)	CRSwNP	IL-4	IL-4 and IL-13 each significantly increase calcium uptake in OSNs *in vitro*.	-	-	IL-4 nasal drops induce anosmia in mice *in vivo*.
Saraswathula et al. (2023)	Conditional expression of IL-13 in mOSNs	IL-13	OSNs are absent in several regions. HBCs are proliferatively active.	Dominant infiltration of macrophage. Modest infiltration of EOSs. Increased mRNA for eotaxin, Muc5AC.	Increased mucin in the Bowman’s glands as well as thick mucin filling the adjacent nasal lumen.	-
Epstein et al. (2008)	AR	Aspergillus fumigatus	Thickness reduction. OSNs apoptosis.	Infiltration of EOSs.	-	-
Ozaki et al. (2010)	AR	Ovalbumin	-	Infiltration of EOSs, macrophages, plasma cells, neutrophils, and mast cells. EOSs especially near nerve fascicles of OSNs. Increased number and size of Bowman’s glands.	-	Impaired.
Carr et al. (2012)	AR	Ovalbumin	Scattered thickness reduction. Swelling glands crowded in ONE. OSNs apoptosis and number loss. GBCs number loss.	Bowman’s glands swell and crowd nerve bundles. Mild EOSs near nerve bundles. Reduced axonal packing of nerve bundles.	-	-
Selvaraj et al. (2012)	AR	Aspergillus fumigatus	Mild inflammation.	EOSs confined to lamina propria and to mucus within the lumen.	Elevated potassium and lower sodium ion concentration in olfactory mucus.	-
Sousa Garcia et al. (2017)	AR in mice with TNFR1 knockout in IOI background	Ovalbumin	Genetic deletion of TNFR1 completely blocks TNF-α-induced inflammation and reduces allergen-induced inflammation. Thinning of ONE in AR, but overall thickness of ONE in IOI-TNFR1^−/−^ mice. Preservation of HBCs in wild type and IOI-TNFR1^−/−^ mice.	Infiltration of EOSs in ovalbumin-sensitized mice. But diminished infiltration of EOSs in ovalbumin-sensitized TNFR1 knockout mice.	-	IOI mice showed a significant reduction in electrical responses, while IOI-TNFR1^−/−^ showed no olfactory loss.

AR, allergic rhinitis; CRS, chronic rhinosinusitis; CRSwNP, chronic rhinosinusitis with nasal polyps; ECRS, eosinophilic chronic rhinosinusitis; ECP, eosinophilic cationic protein; EOSs, eosinophils; GBCs, globose basal cells; HBCs, horizontal basal cells; imOSNs, immature olfactory sensory neurons; IOI, inducible olfactory inflammation; mOSNs, mature olfactory sensory neurons; ONE, olfactory neuroepithelium; OSNs, olfactory sensory neurons; TNF-α, tumor necrosis factor α; TNFR1, tumor necrosis factor receptor 1; TSLP, thymic stromal lymphopoietin.

**Figure 1:**
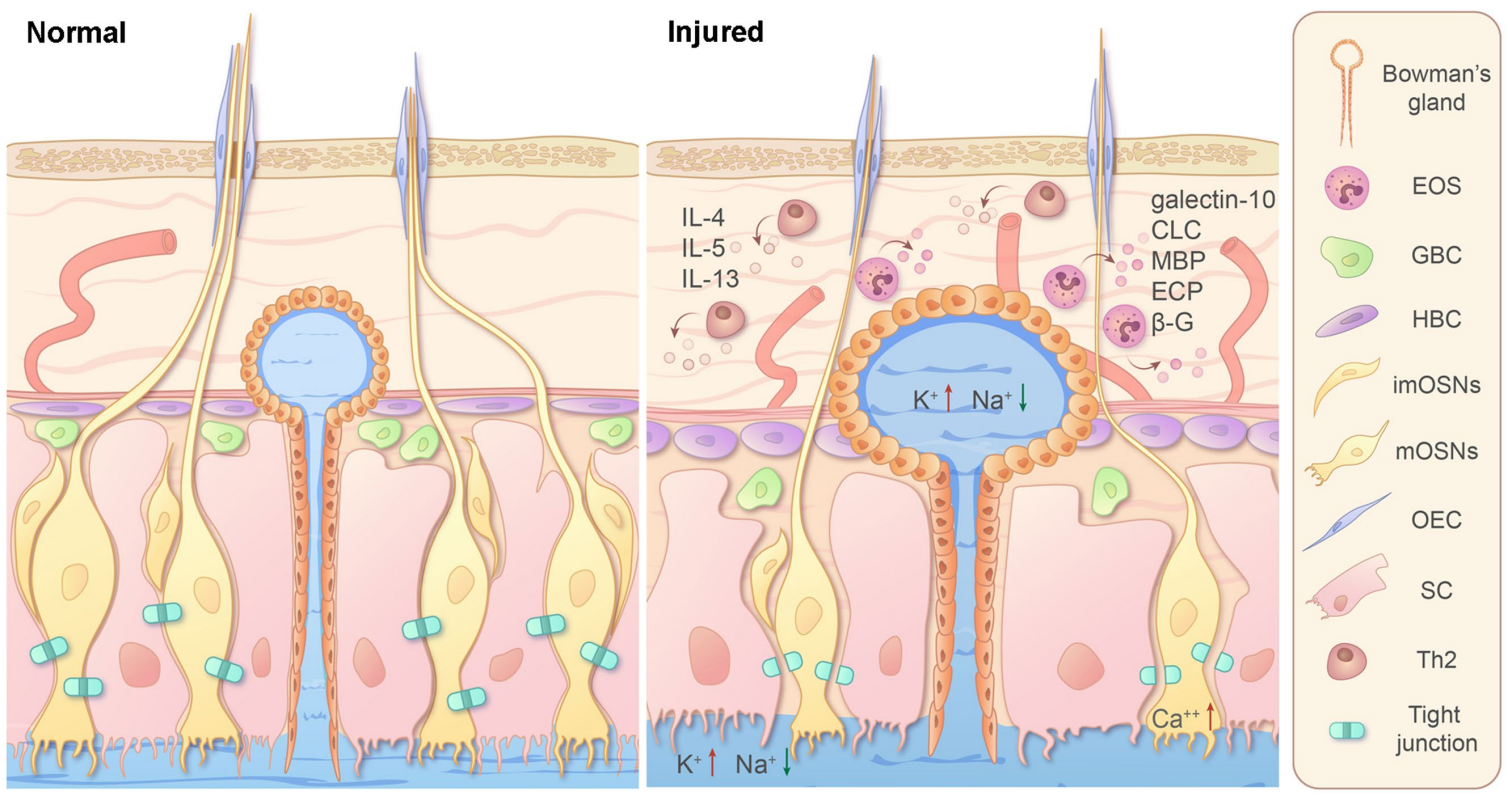
Schematic diagram illustrating the mechanisms of EOS-induced injury of OM with experimental inflammation in murine models. Infiltration of EOSs around neural axons, eosinophil granules, and type 2 cytokines in lamina propria leads to structural changes in OM, number loss of mature OSNs, immature OSNs, and GBC, a slightly increased number of HBC, and ion concentration alterations in OSNs and mucus. ECP, eosinophilic cationic protein; EOS, eosinophil; GBC, globose basal cell; HBC, horizontal basal cell; imOSNs, immature olfactory sensory neurons; MBP, major basic protein; mOSNs, mature olfactory sensory neurons; OEC, olfactory ensheathing cell; SC, sustentacular cell; Th2, T helper 2 cell; β-G, β-glucuronidase.

In OM with induced inflammation, significant eosinophilic infiltration is limited in edematous lamina propria and crowds neural axons together with hypertrophied Bowman’s glands, accompanied by thinned ONE, number loss of mature OSNs, immature OSNs, and globose basal cells (GBCs), slightly increased number and proliferative activity of horizontal basal cells (HBCs), loss of tight junction between OSNs and sustentacular cells, increased calcium in OSNs, and elevated potassium and lower sodium ion concentration in thick olfactory mucus (Figure 1).

EOSs may induce OSN apoptosis through axonal disruption rather than direct neuronal cell body damage above the basement membrane. First, progressive eosinophilic infiltration around the axon and subsequent edema in lamina propria compresses axonic bundles of OSNs (Ozaki et al., 2010; Carr et al., 2012; Sousa Garcia et al., 2017). Second, eosinophil granules induce axonal degeneration (Lavin et al., 2017). Third, eosinophilic infiltration, Th2 cells, and type 2 inflammation such as IL-13 induce hypertrophy and an increased number of Bowman’s glands in lamina propria crowd olfactory axonic bundles (Ozaki et al., 2010; Carr et al., 2012; Saraswathula et al., 2023). Hypersecretion of Bowman’s glands with high potassium and low sodium ion concentrations in the olfactory mucus may also contribute to reducing the sense of smell through impairing diffusion of olfactants and affecting the microenvironment and transduction activity of OSNs (Selvaraj et al., 2012).

Direct damage to the neural body may also cause OSN apoptosis and dysfunction. First, IL-4 and IL-13 can significantly increase calcium uptake of murine OSN *in vitro* and cause neural dysfunction. IL-4 nasal drops can induce anosmia *in vivo* (Hara et al., 2023). Second, conditional expression of IL-13 in mature OSNs and consequent type 2 inflammation-induced alterations in tight junctions in sustentacular cells can injure the barrier function of ONE and finally affect neuronal survival and function (Saraswathula et al., 2023). However, the molecular mechanisms of EOSs and type 2 inflammation-induced apoptosis and dysfunction of OSNs have not yet been well elucidated.

OSNs with functional changes may recover as early as day 3 of treatment with anti-IL-4Rα antibody dupilumab in patients with difficult-to-treat CRSwNP (Mullol et al., 2022). This recovery is quicker than the maturation of newborn ONSs, which may take 8–10 days for the axons to target the OB (Liberia et al., 2019). Dupilumab is effective in reducing nasal eosinophilic infiltration (De Corso et al., 2022). Transient increase in blood eosinophil count after dupilumab treatment in patients with CRSwNP does not affect its effect on clinical symptoms (Wechsler et al., 2022). IL-4Rα, receptor of IL-4 and IL-3, is expressed in mature OSNs as well as in HBCs and GBCs, which implies direct anti-inflammation of dupilumab and that biological therapy may have direct modulation on neuroimmune interaction on ONE in addition to reduced polyp size, mucosal edema, and symptom severity (Colquitt et al., 2013; Hara et al., 2023; Kim et al., 2023). Anti-IgE antibody omalizumab and anti-IL-5 antibody mepolizumab are proven to improve olfactory function and decrease peripheral eosinophil counts and eosinophilic infiltration in sinonasal mucosa and OM (Le et al., 2018; Gevaert et al., 2020; Tiotiu et al., 2020; Walter et al., 2022).

Intranasal and oral corticosteroids are currently the mainstay treatment for ECRSwNP, as they may induce apoptosis and reduce the number of EOSs (Watanabe et al., 2004). The olfactory function can recover rapidly by reducing the size of nasal polyps and inflammatory burden in both the sinonasal mucosa and OM, and long-term recovery is expected by rebuilding the structure of the OM. However, the effect of short-term use of corticosteroids has a lasting effect on nasal symptoms, including olfactory function, for only 8–12 weeks and may require repeated use of corticosteroids (Banglawala et al., 2014). Furthermore, there are unexpected findings that topical corticosteroids may reduce olfactory function by inducing neuronal apoptosis and OSN turnover disturbance (Takanosawa et al., 2009; Crisafulli et al., 2018). In an allergic rhinitis mouse model, intranasal fluticasone propionate treatment improves olfactory function and reduces EOS-associated inflammation in OM but fails to rebuild a mature OSN population (Li et al., 2023).

HBCs normally differentiate into GBCs, which continuously differentiate into OSNs and sustentacular cells at a turnover rate of 30–90 days (Schwob et al., 2017). Inflammation within the OM can lead to temporary and reversible OSN turnover disruption. In animal models with EOS-related inflammation in OM, there is an inconsistency between the number of loss of immature OSNs and GBCs and a slight increase in the number and proliferative activity of HBCs (Saraswathula et al., 2023). In an inducible olfactory inflammation mouse model with TNFα expression in sustentacular cells, HBCs lose stem cell characteristics and the ability of OSN regeneration and switch to a phenotype that participates in immune recruitment and modulation through the NF-κB-mediated pathway. Following the cessation of TNFα-induced inflammation, HBCs can switch back to a regenerative phenotype and replace lost OSNs (Chen et al., 2019). However, the molecular mechanisms of turnover disruption of OSNs and the changed function of HBCs in ECRS remain unknown.

Loss of axonal connection between OSNs and OB not only leads to the apoptosis of OSNs but also leads to the apoptosis of relay neurons within OB. Slight or moderate OSN loss without detectable OD in patients with CRS can lead to reduced OB volumes (Rombaux et al., 2008). Results from the murine OD model imply that the decline of olfactory function can be detected only when OSN loss reaches a certain level (Li et al., 2023). Changes in OB size are more sensitive than OD in detecting OSN loss in patients with CRS. Magnetic resonance imaging scans show that along with severe OD, patients with CRSwNP have a significant reduction of volume in OB and gray matter in olfactory brain regions (Herzallah et al., 2013; Han et al., 2017). Axonal connection with targeting glomeruli cells in OB is a determinative process for the maturation of newborn OSNs (Liberia et al., 2019). Increased volume in both the OB and gray matter of primary and secondary olfactory cortices can be observed following olfactory function recovery after surgical treatment (Gudziol et al., 2009; Whitcroft et al., 2018).

EOSs secrete not only various chemokines, cytokines, and neurotoxins that may lead to neuronal damage but also neurotrophins (Wu et al., 2023). There are direct connections between SP-positive nerve fibers and EOSs in atopic dermatitis. EOS-released neurotrophins may induce the branching of cultured neurons (Guseva et al., 2020). EOSs and type 2 cytokines may have a promotional role in peripheral nerve regeneration across a nerve gap injury. On the other hand, loss of EOSs and type 2 cytokines may delay axon regeneration and functional recovery in a segmental nerve injury model (Pan et al., 2021; Liebendorfer et al., 2023). Although neurotrophins are proinflammatory mediators, it is reported that in the sinus mucosa of patients with CRS, the concentration of nerve growth factor increases while the concentration of brain-derived neurotrophic factor decreases (Coffey et al., 2009). However, the favorable roles of EOSs in the turnover of OSNs have not been defined yet.

## Conclusion

Tissue eosinophilia is closely associated with morphological damage to OM and OD in patients with ECRS, independent of polyps and disease severity. In the OM of patients with ECRS, only EOSs have an adverse impact on the integrity of the ONE and are correlated with mucosal erosion, loss of OSNs, and OD. EOSs can lead to the apoptosis of OSNs through axonal and neural body damage, turnover disorder of OSNs through the loss of immature OSNs and GBCs and changed proliferative activity of HBCs, and dysfunction of OSNs through the breakdown of neuroepithelial integrity and alteration of ion concentration in OSNs and mucin. Treatment with biotherapeutics, corticosteroids, and surgery may recover olfactory function by reducing eosinophilic infiltration in the OM. However, the molecular mechanisms of tissue eosinophilia-induced apoptosis and turnover disruption of OSNs in patients with ECRS have not been well elucidated. Further investigations are warranted to obtain new therapeutic targets and achieve better restoration of olfactory function.

## Author contributions

YC: Writing – original draft, Writing – review & editing. ML: Writing – original draft. JL: Writing – original draft, Writing – review & editing.
